# Numerical and experimental validation of Taguchi-optimized star-core fin inserts for enhanced thermal hydraulic performance factor

**DOI:** 10.1038/s41598-025-31597-8

**Published:** 2025-12-12

**Authors:** Zeki Ali Al-Saadi, Adnan Ibrahim, Sharul Sham Dol, Firas Abdulamir Radhi, Hariam Luqman Azeez, Anwer B. Al-Aasam, Norasikin Ahmad Ludin, Ahmad Fazlizan

**Affiliations:** 1 Solar Energy Research Institute, Universiti Kebangsaan Malaysia, Bangi, 43600 Selangor Malaysia; 2 Department of Mechanical and Industrial Engineering, Abu Dhabi University, PO Box 59911, Abu Dhabi, United Arab Emirates; 3 Department of Mechanical Power Engineering, Technical Engineering College, Middle Technical University, Baghdad, Iraq

**Keywords:** Fin inserts, Taguchi optimization, Experimental validation, Heat transfer, Thermo-hydraulic performance factor, Energy science and technology, Engineering, Mathematics and computing

## Abstract

Enhancing heat transfer in compact thermal systems remains a key engineering challenge, where internal geometry plays a decisive role in disrupting flow, increasing surface exposure, and boosting convective efficiency. In this study, a novel star-core fin insert was developed, featuring angularly spaced radial fins mounted on a central rod to improve the thermal-hydraulic performance factor (THPF). The design was optimized using a hybrid approach that combined the Taguchi method with Computational Fluid Dynamics (CFD) simulations, enabling systematic evaluation of five geometric parameters: fin diameter, number of fin edges, number of fins per rod, fin thickness, and angular offset. These parameters were varied concurrently to capture interaction effects and identify the most effective configuration. The optimal configuration (Case 31) achieved a THPF of 1.75, representing a 75% improvement over the smooth pipe baseline, with a fin diameter of 14.5 mm, five edges, four fins per rod, a thickness of 3 mm, and a 0° angular offset. A physical prototype of the absorber tube was fabricated and tested experimentally, showing strong agreement with numerical predictions (correlation = 95.5%, RMSE = 4.5%). These findings demonstrate that the integrated optimization framework is reliable and effective, improving heat transfer performance while maintaining acceptable pressure losses. The proposed passive design offers a scalable and energy-efficient solution for next generation compact heat exchangers.

## Introduction

Heat exchangers are fundamental to numerous engineering sectors, including HVAC, chemical and petrochemical processing, power generation, refrigeration, electronics cooling, and photovoltaic thermal (PVT) systems, where controlled thermal-energy exchange improves efficiency, reliability and component lifetime^1^. Thermal management in photovoltaic (PV) devices is a critical matter: high cell temperature decreases electrical conversion efficiency, increases thermal losses, and degrades the material used^2^, and therefore combining efficient methods of heat-extraction with PV modules has the potential to increase electrical performance and operational lifespan^3,4^. Water continues to be the most common working fluid in PV thermal (PVT) systems due to its universal nature, high specific heat capacity and desirable thermal transport characteristics, in combination with properly designed channels and surface enhancements, water can be able to remove heat in a realistic installation^5^ A large amount of experimental and numerical literature has shown that purposeful geometrical changes and internal inserts can significantly increase the convective heat transfer, but often with a burden associated with pressure and drop across the resistance to flow. As an example, variable-step twisted spiral and elliptical tubes can be used to promote cross-stream mixing and streamline re-alignment and thus the coefficient of heat-transfer and the uniformization of temperature fields^6^ At smaller scales, surface texturing has produced significant local gains: Liao and Jing^7^ reported up to a 38% enhancement in local heat transfer for 1–2 mm dimple protrusions at Re 5 000, showing that modest surface perturbations can energize near‑wall flow and disturb thermal boundary layers.

Careful dimple design yields thermodynamic benefits as well; Kaood et al.^8^ observed a 7.8% increase in the thermal-hydraulic performance factor (THPF) and a 36.9% reduction in entropy generation using stepped conical dimples in convergent tubes. Insert and otherwise surf texturing can be utilized in combination to achieve synergies. The dimple configurations coupled with twisted tapes are better than the traditional tapes under low Reynolds numbers, whereby secondary vortices and enhanced mixing dominate heat transport^9^. Similarly, dimpled twisted‑tape arrangements in conjunction with SiO2 nanofluids have been shown to increase Nusselt numbers substantially in selected parametric regimes^10^. Aggressive insert geometries may yield very large heat‑transfer gains but at the cost of hydraulic losses: Singh and Kumar^11^reported a 123.6% increase in Nusselt number for triangular perforated twisted tapes, accompanied by an approximately 495% increase in friction factor, illustrating the fundamental trade‑off between thermal enhancement and pumping burden. These trade-offs can be reduced through design optimisation, Abdulhamed et al.^12^ have shown that optimization of perforation density and geometry can generate 97% increases in Nu, as well as 89% improvements in THPF over baseline designs.

The coupling of structured inserts with advanced working fluids has extended the achievable performance envelope. Twisted geometries operating with Al2O3, CuO, or other nanoparticle suspensions have yielded heat‑transfer improvements exceeding 100% in certain studies, underlining the potential of fluid‑geometry synergy^13,14^. Hybrid PVT collectors combining twisted‑tape inserts and nanofluids have reported THPF values approaching 1.37 in experimental configurations, signaling notable gains in combined thermal and electrical performance^15^. Micro‑fin absorber tubes, spring or coil inserts, and integrations with nanoparticle‑enhanced phase‑change materials (nano‑PCM) have likewise demonstrated improvements in thermal regulation and energy‑storage capacity^16,17^. Nevertheless, several practical challenges constrain the widespread adoption of nanofluids and nano‑PCM: long‑term stability and particle agglomeration, increased viscosity and pumping energy, handling complexity, higher cost, and environmental or maintenance concerns^16,18^. Moreover, many high‑performing geometries impose substantial hydraulic penalties that can negate net system benefits in compact or low‑power applications^14,19^. These limitations motivate the pursuit of purely geometric, passive strategies that achieve meaningful hydrothermal improvement while minimizing hydraulic cost and avoiding potentially unsustainable additives.

Efficient exploration of the high‑dimensional design space that governs geometric augmentations is therefore essential. The Taguchi method a design of experiments (DOE) framework frequently combined with grey relational analysis or response‑surface techniques permits systematic assessment of multiple geometric and operational factors (for example, dimple concavity, twisted‑tape pitch and thickness, fin geometries, Reynolds number and flow rate) using a reduced number of trials, enabling identification of near‑optimal configurations with modest experimental or computational expense^12,20^. Prior heat‑exchanger and PVT studies employing Taguchi‑type frameworks have successfully extracted robust parameter settings and clarified factor significance across large parameter ranges, thereby guiding efficient refinement and resource allocation^21^.

Despite these advances, a research gap persists: many high‑performance concepts remain fixed geometry, rely on nanomaterials or PCMs, or impose unacceptable hydraulic penalties that limit practical deployment in PVT thermal management and compact heat‑exchanger systems^18,22^. Additionally, manufacturability, scalability, and long‑term durability of complex geometries are often under‑addressed, hindering translation from laboratory demonstration to field implementation. Recent studies that combine geometric innovation with advanced fluids have shown promise but inherit the practical drawbacks of the fluids themselves and, in some cases, increased operational complexity^17,23^.

To address this gap, the present work proposes and evaluates a novel star‑shaped finned absorber tube: a star‑core fin insert mounted on a central copper rod, with rod and fins fabricated from copper to ensure high thermal conductivity and structural robustness. The principal novelty lies in a systematic parametric investigation of star‑fin geometry including the number of star points (edges), fin (fan) diameter, number of fins per rod, phase angle (angular offset) between adjacent fins on the same axis, and fin thickness aimed at generating controlled vortical structures, disrupting thermal boundary layers and increasing effective heat‑transfer area while preserving manufacturability and strictly passive operation^18,19^. The design focuses on a variable geometry design to enable on-site tuning and scalability, overcoming constraints posed by fixed-geometry inserts, and relying on nanoparticulate additives, it does not depend on nanoparticulate additives.

The combined Taguchi DOE with the computational fluid dynamics (CFD) simulation is used to investigate the multidimensional design space effectively and measure the trade-off between convective heat-transfer augmentation and hydraulic burden. The main aims of the research are as follows: (i) measure the hydrothermal performance of the star core fin over a realistic range of Reynolds numbers, (ii) determine near-optimal combinations of the geometry parameters using Taguchi analysis, and (iii) compare the optimized performance of the star core with the smooth and selected augmented inserts in terms of Nusselt number, friction factor, and THPF. The solution will involve the combination of a physics-based simulation with a statistically effective DOE to determine the star-fin designs that will improve thermal harvesting and optimize the rate of heat exchange in small exchangers and maintain low pumping demands. The proposed idea aims to offer a scalable alternative to PCM or nanofluid-dependent methods that will offer meaningful performance gains at a lower environmental cost.

## Methodology

### Numerical implementation and design strategy

**Fig. 1:**
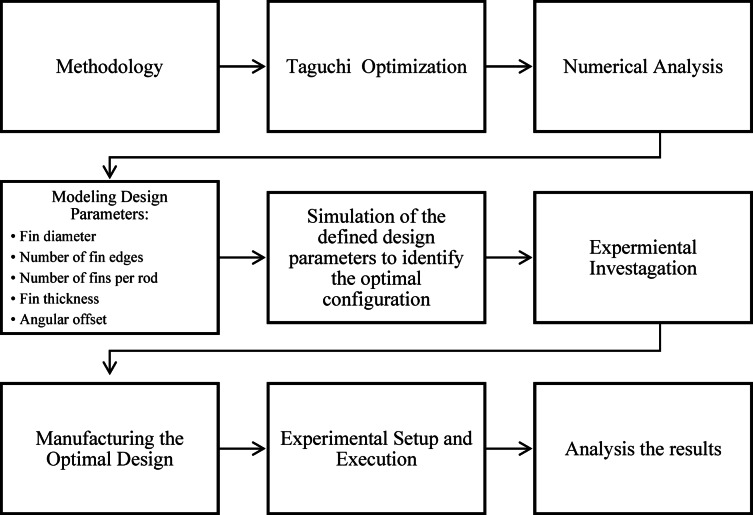
Overview of methodology.

This study presents an integrated methodology for enhancing THPF in tubular heat exchangers, with direct applicability to PVT modules, chemical reactors, and compact energy recovery systems. The methodological flow chart is provided in Fig. 1. It shows the process followed on the absorber tube whereby there is a stationary central rod with the inserts of star cores at fixed positions, whose geometry is governed by five key parameters: number of edges (E), fin diameter (D), fin thickness (T), number of fins per rod (F), and angular spacing (A). These parameters are summarized in Table 1. The angular offset represents the rotational phase difference between consecutive star‑core fin plates mounted along the central rod. In practical terms, it is the angle by which one star is rotated relative to the next star on the same rod. A 0° offset means all plates are aligned in the same orientation, while a non‑zero offset produces a staggered or helical arrangement. This distinction ensures that “angular spacing” refers to the distribution of edges within a single plate, whereas “angular offset” describes the relative rotation between successive plates.

Each configuration was evaluated using CFD simulations within a Reynolds number range from 6,000 to 15,000. The simulations provided detailed insights into vortex formation, pressure drop, and thermal distribution, critical metrics for assessing THPF and guiding design decisions.

The optimization process followed three iterative phases:

- Design exploration: Parameters were introduced into MATLAB, and a Taguchi L18 orthogonal array was applied to generate 18 baseline configurations. To ensure comprehensive coverage, an additional 14 cases were added, resulting in 32 candidate designs. All geometries were subsequently modelled in AutoCAD for numerical analysis by ANSYS 2025 R1.
- Prototype fabrication: The optimal configuration was manufactured using precision laser machining to ensure fidelity with simulation outputs.
- Experimental validation: The prototype was tested under controlled laboratory conditions and benchmarked against CFD predictions to validate the numerical model.

This integrated framework, combining simulation, optimization, and experimental verification, offers a reproducible pathway for passive cooling enhancement in industrial applications.

**Table 1:** Geometric parameters for star-core fin inserts.

Parameter	Description	Range /values
No. of Edges	E	2–5
Fin diameter (mm)	D	14.0- 15.5
Fin thickness (mm)	T	2 -3.5
Number of fins per rod	F	4–8
Angular spacing between fins (°)	A	0–40°
Copper Pipe length	550 (mm)	
Outer Diameter	19.10 (mm)	
Inner Diameter	16.96 (mm)	
Rod Diameter	4 (mm)	
Rod length	550 (mm)	

### Taguchi-Based design optimization

To reduce computational overhead and accelerate convergence toward optimal designs, a Taguchi‑based design of experiments (DOE) methodology was employed. Five geometric parameters were identified as most influential on internal surface area and thermal hydraulic performance. These were systematically varied using an L18 orthogonal array, which efficiently captures main effects while reducing the number of required simulations compared to full factorial designs. An additional 14 cases were added, resulting in 32 candidate designs.

To enhance robustness and explore potential parameter interactions, the initial 18 configurations were expanded to 32 design cases. Each configuration was modeled in AutoCAD and evaluated through CFD simulations in ANSYS Fluent^20^.

### CFD simulation

CFD simulations were conducted using ANSYS Fluent 2025 to evaluate THPF of all 32 configurations derived from the Taguchi matrix. To accurately resolve flow structures induced by internal fins and localized vortices, four turbulence models were assessed: RNG k-ε, Realizable k-ε, Reynolds Stress Model (RSM), SST k-ε, and the Realizable k-ε model with enhanced wall treatment was selected for its superior predictive accuracy and computational efficiency, particularly in capturing near-wall gradients and complex flow interactions^24^.

Boundary conditions included:

- Uniform surface heat flux: 1000 W/m^2^.
- Ambient temperature: 25 °C.
- Operating pressure: 1 atm.
- Working fluid: Water with constant thermophysical properties^5^.
- No-slip conditions at all solid-fluid interfaces.

Mesh quality was validated through a grid independence test. The final mesh comprised approximately 293,786 cells and 1.78 million interior faces, achieving a minimum orthogonal quality of 0.2 and a maximum aspect ratio of 10.13, with no isolated cells detected. Nusselt number stabilized beyond 250,000 elements, confirming mesh independence and ensuring reliable predictions of friction factor and Nusselt number. Near-wall resolution was enhanced by maintaining appropriate y⁺ values, enabling accurate capture of boundary layer phenomena.

**Fig. 2:**
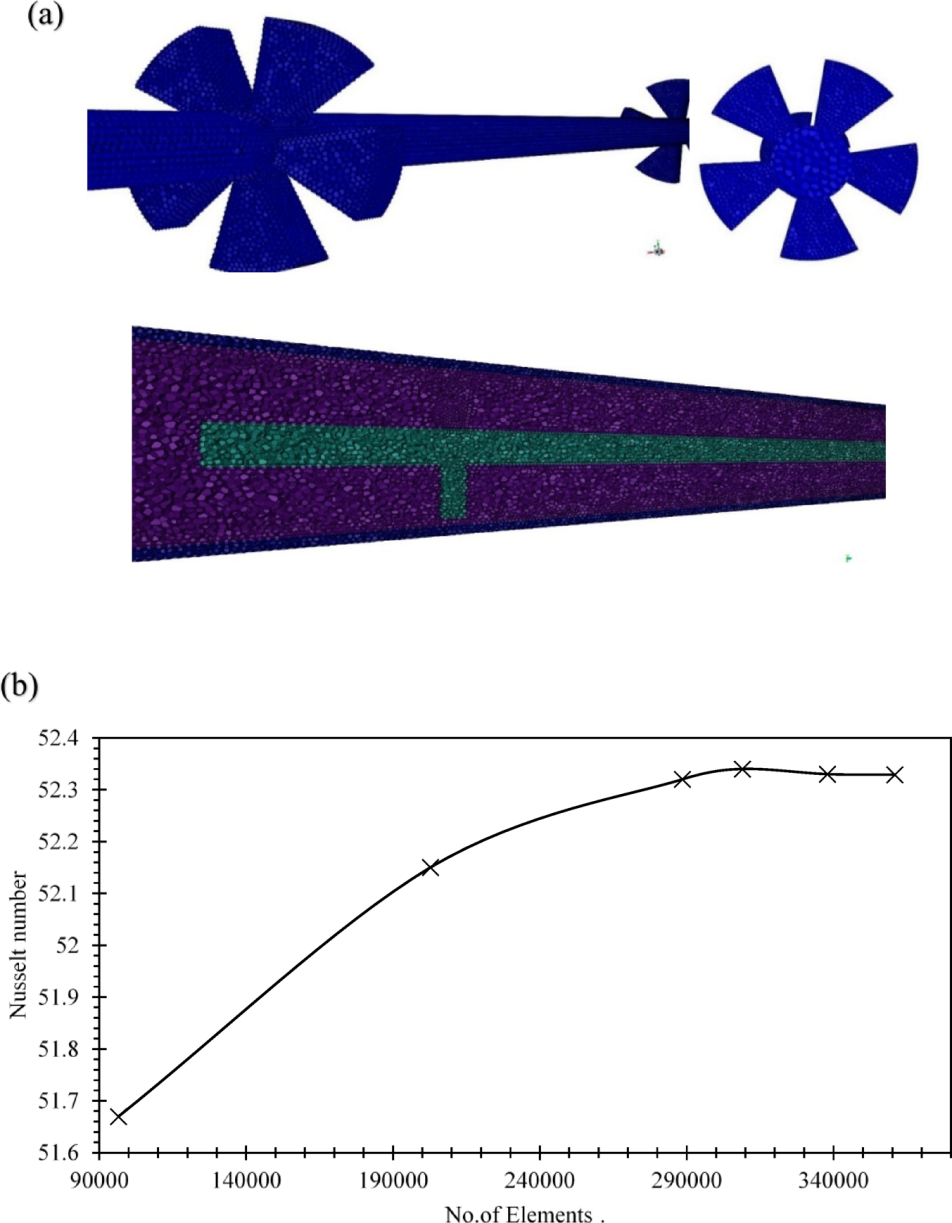

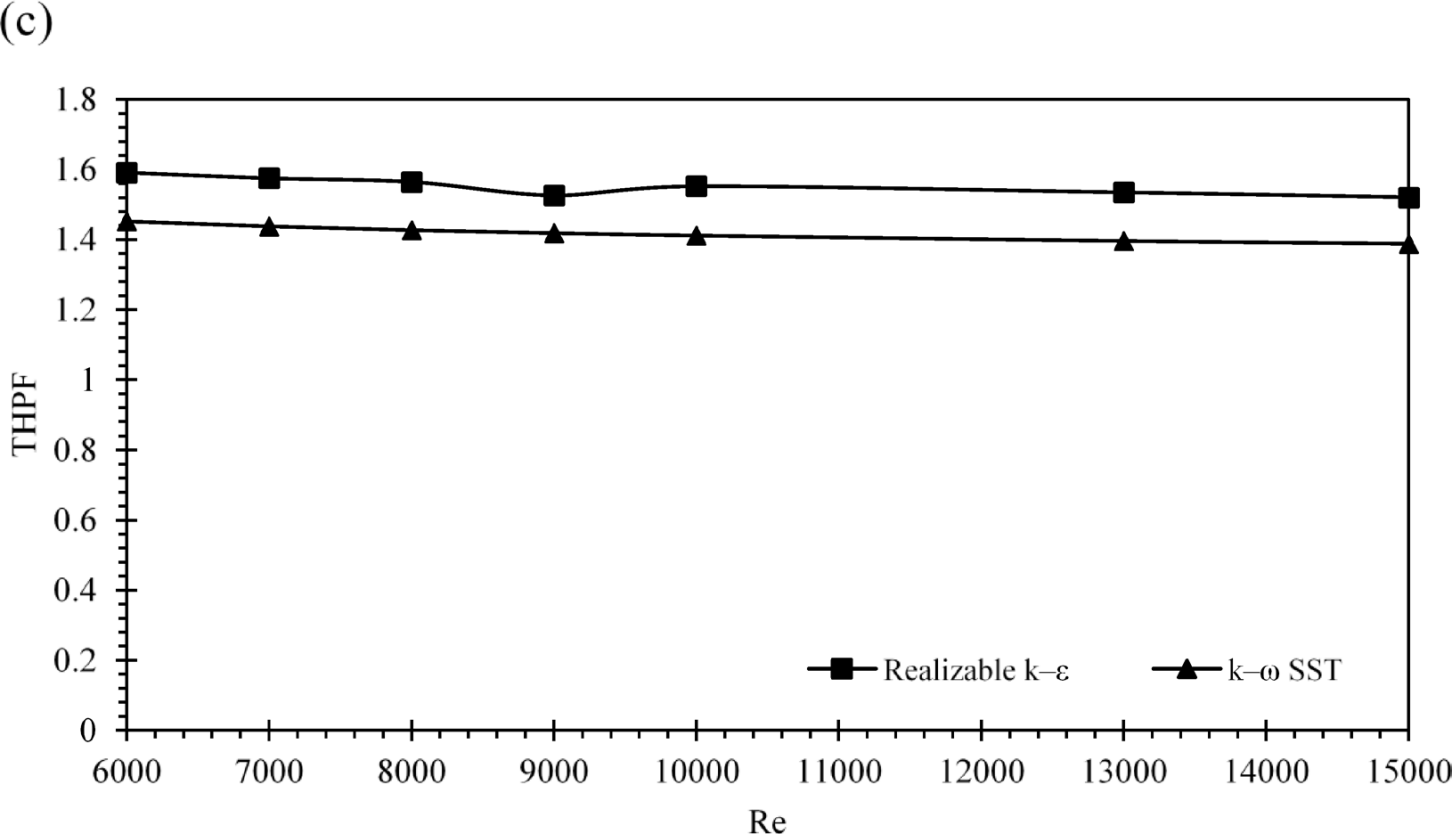
Mesh Quality and Configuration Analysis: (**a**) Cross-Sectional View, 3D Meshing for fins insert, (**b**) Grid independence Test, (**c**) Comparison of Thermal-Hydraulic Performance Factor (THPF) Between Realizable k-ε and k-ω SST Models.

Figure 2a presents a cross-sectional view of the meshed domain, highlighting the geometric complexity of the finned region. Figure 2b illustrates the convergence behavior of the Nusselt number during grid refinement. Together, these visualizations confirm the robustness of the numerical setup and the fidelity of the mesh in capturing critical flow features.

In Fig. 2c the Realizable k-ε model exhibits a consistently higher performance value, remaining slightly above 1.5 throughout the tested range. The k-ω SST model shows slightly lower values, remaining just below 1.5, with minimal deviation. This comparative trend suggests that, for the specific flow configuration and geometry studied, the Realizable k-ε model provides marginally better predictive stability and aligns well with experimental observations. While the k-ω SST model is known to capture near-wall and vortex dynamics more accurately, the flow regime in our case did not exhibit strong swirl or separation zones that would necessitate SST or Reynolds Stress Model complexity.

Therefore, the Realizable k-ε model was selected for its balance between computational efficiency and predictive accuracy. We acknowledge the limitations of this choice and have included this comparative analysis to justify the modeling approach.

### Physical model fabrication

**Fig. 3:**
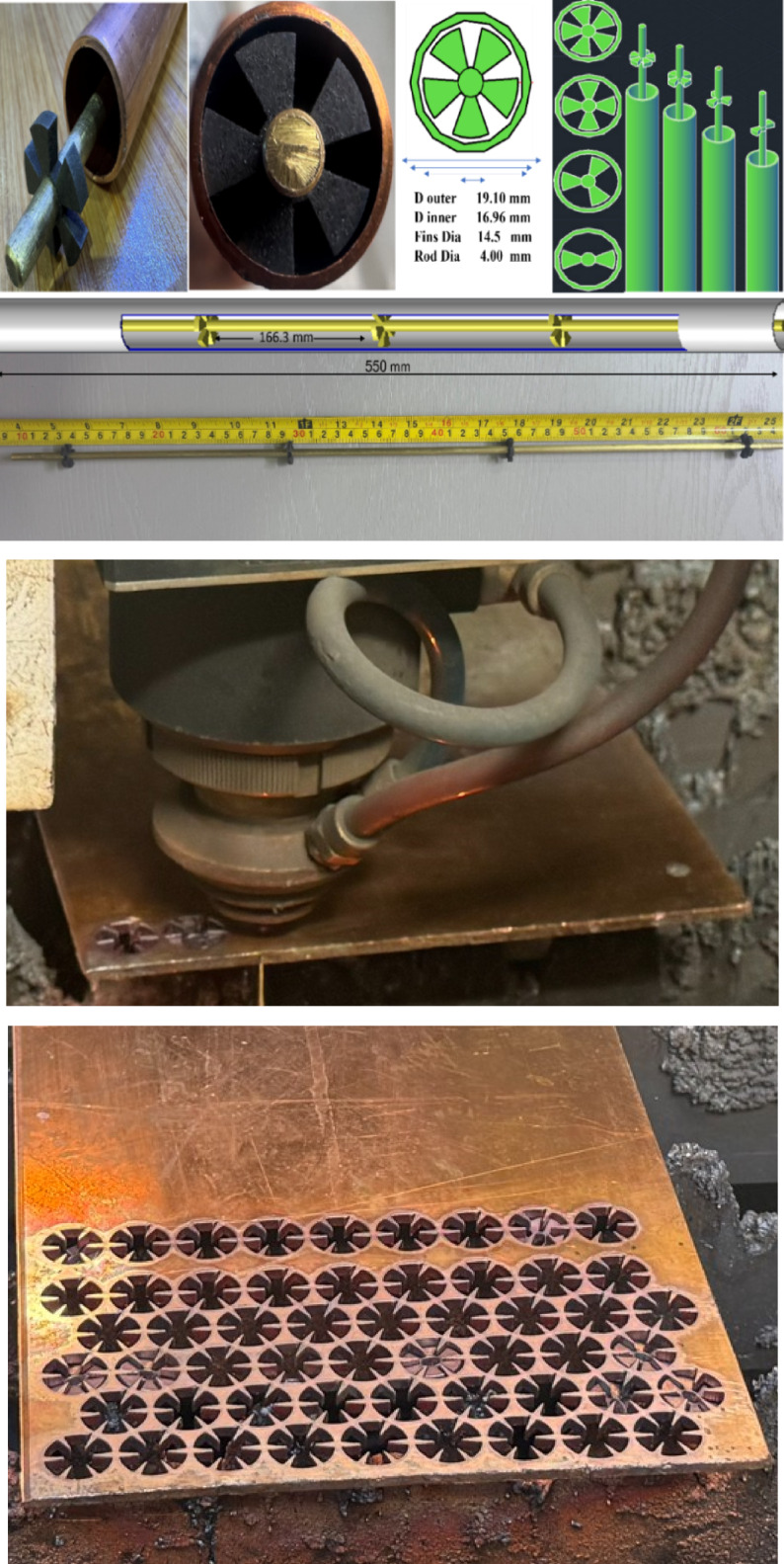
Dimensional layout and physical model used in both simulation and experimental phases.

The absorber tube was fabricated from high-conductivity copper to ensure efficient thermal performance in compact heat exchanger applications. It features an outer diameter of 19.10 mm, an inner diameter of 16.96 mm, and a total length of 550 mm. A stationary central rod of 4.00 mm diameter was inserted along the tube axis, onto which cross-shaped fin elements were mounted with a diameter of 14.5 mm and a thickness of 3.00 mm, as shown in Fig. 3.

The fin configuration precisely reflects the optimized geometry derived from CFD simulations, based on the five key design parameters. Configurations outside the defined range were excluded due to fabrication constraints, meshing complexity, and diminishing thermal returns. The physical prototype was fabricated without additional geometric modifications, ensuring complete alignment with the simulated model and preserving both manufacturability and thermal-hydraulic integrity.

### Performance metrics and evaluation

To assess the thermo-hydraulic behavior of each configuration, the following performance indicators were computed:

- Friction factor (f).
- Surface heat transfer coefficient (h).
- Nusselt number (Nu).
- Thermal-Hydraulic Performance Factor (THPF), benchmarked against a smooth tube reference.

All simulations and post-processing were conducted using Ansys and MATLAB, employing automated scripts and structured data arrays to ensure consistency and reproducibility. Visualization tools, including surface plots, 3D mesh networks, and comparative bar charts, were used to analyze the influence of geometric variations and flow conditions on THPF.

### Governing equations

Hydrodynamic and thermal fluid behavior within the system was analyzed using CFD, grounded in the core conservation laws of fluid mechanics. These include the physical laws dictating mass, momentum, and energy transfer, which collectively govern the transport phenomena across the computational domain. The mathematical formulation, presented in Eq. (1) through (6) and referenced in the Nomenclature section, defines the simulation framework. Central to this formulation is the continuity equation, which ensures mass conservation by preventing the generation or loss of mass within the control volume. This principle serves as the basis for constructing the momentum and energy equations. It forms the foundational principle upon which the momentum and energy equations are constructed^25^.

Conservation of mass.

1 $$\:\frac{{\partial \:\left( {\rho \:{\mathrm{u}}_{{\mathrm{i}}} } \right)}}{{\partial \:{\mathrm{x}}_{{\mathrm{i}}} }} = 0$$

Conservation of momentum

2 $$\:\frac{\partial\:}{\partial\:{\mathrm{x}}_{\mathrm{i}}}\:{\uprho\:}{\mathrm{u}}_{\mathrm{i}}{\mathrm{u}}_{\mathrm{j}}\:=\:\frac{\partial\:\mathrm{p}}{\partial\:{\mathrm{x}}_{\mathrm{i}}}\:+\frac{\partial\:}{\partial\:{\mathrm{x}}_{\mathrm{j}}}\left[\right({\upmu\:}+{{\upmu\:}}_{\mathrm{t}}\left)\right(\frac{\partial\:{\mathrm{u}}_{\mathrm{i}}}{\partial\:{\mathrm{x}}_{\mathrm{j}}}+\frac{\partial\:{\mathrm{u}}_{\mathrm{i}}}{\partial\:{\mathrm{x}}_{\mathrm{i}}})-\frac{2}{3}{{\updelta\:}}_{\mathrm{j}\mathrm{i}}({\upmu\:}+{{\upmu\:}}_{\mathrm{t}}\frac{\partial\:{\mathrm{u}}_{{\uplambda\:}}}{\partial\:{\mathrm{x}}_{{\uplambda\:}}}\left)\right]$$

Conservation of energy

3 $$\:\frac{\partial\:}{\partial\:{\mathrm{x}}_{\mathrm{i}}}\:{\uprho\:}{\mathrm{u}}_{\mathrm{i}}\mathrm{T}\:=\:\frac{\partial\:}{\partial\:{\mathrm{x}}_{\mathrm{i}}}\:(\frac{{{\upmu\:}}_{\mathrm{t}}}{{\mathrm{P}\mathrm{r}}_{\mathrm{T}}}\:+\frac{{\upmu\:}}{\mathrm{P}\mathrm{r}})\frac{\partial\:\mathrm{T}}{\partial\:{\mathrm{x}}_{\mathrm{i}}}\:$$

Turbulent kinetic energy (k)

4 $$\:\frac{\partial\:}{\partial\:{\mathrm{x}}_{\mathrm{i}}}\left({\uprho\:}{\mathrm{u}}_{\mathrm{i}}\mathrm{k}\right)=\:\frac{\partial\:}{\partial\:{\mathrm{x}}_{\mathrm{i}}}\left[\right({\upmu\:}\:+\frac{{{\upmu\:}}_{\mathrm{t}}}{{{\upsigma\:}}_{\mathrm{k}}}\left)\frac{\partial\:\mathrm{k}}{\partial\:{\mathrm{x}}_{\mathrm{i}}}\right]+{\uprho\:}{\Gamma\:}-{\uprho\:}{\upvarepsilon\:}$$

Specific heat dissipation rate $\:\left({\upvarepsilon\:}\right)$

5 $$\:\frac{\partial\:}{\partial\:{\mathrm{x}}_{\mathrm{i}}}\left({\uprho\:}{\mathrm{u}}_{\mathrm{i}}{\upvarepsilon\:}\right)=\frac{\partial\:}{\partial\:{\mathrm{x}}_{\mathrm{i}}}\left[\right({\upmu\:}\:+\frac{{{\upmu\:}}_{\mathrm{t}}}{{{\upsigma\:}}_{{\upvarepsilon\:}}}\left)\frac{\partial\:{\upvarepsilon\:}}{\partial\:{\mathrm{x}}_{\mathrm{i}}}\right]+{\mathrm{c}}_{1}{{\Gamma\:}}_{{\upvarepsilon\:}}-{\mathrm{c}}_{2}\frac{{{\upvarepsilon\:}}^{2}}{\mathrm{k}+\sqrt{\mathrm{v}{\upvarepsilon\:}}}$$

6 $$\:\Gamma \: = - \bar{u}_{i} \bar{u}_{j} \frac{{\partial \:u_{i} }}{{\partial \:u_{j} }} = \frac{{\mu \:_{t} }}{\rho }\left( {\frac{{\partial \:u_{i} }}{{\partial \:x_{j} }} + \frac{{\partial \:u_{i} }}{{\partial \:x_{i} }}} \right)\frac{{\partial \:u_{i} }}{{\partial \:x_{j} }}$$

### Experimental rig

**Fig. 4:**
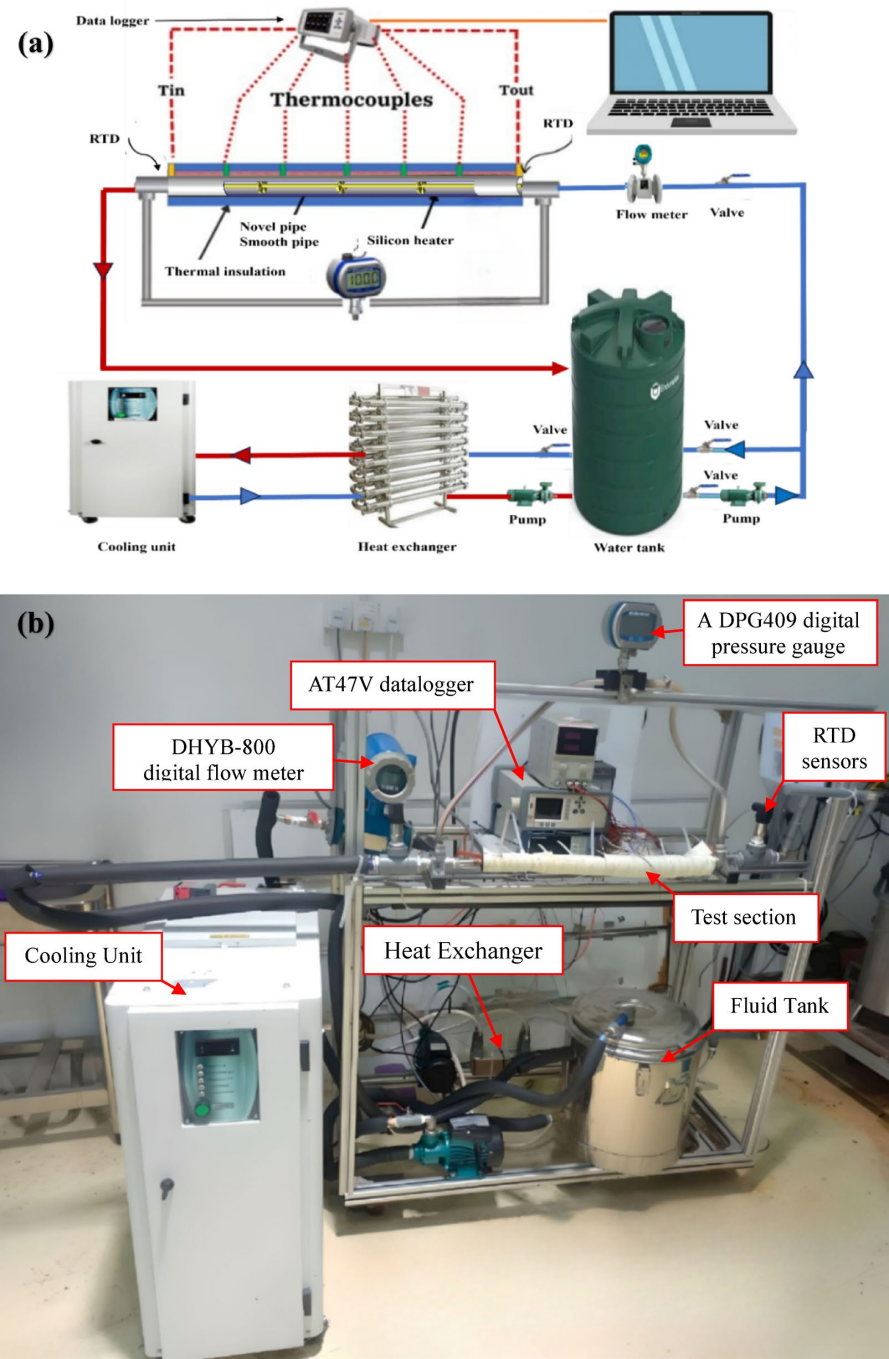
(**a**) Conceptual illustration depicting the system layout and (**b**) experimental apparatus.

The experimental campaign was initiated using water as the working fluid in a smooth pipe, which was subsequently replaced by a novel fin-core insert design (Fig. 4). Tests were conducted under a constant heat flux of 1000 W/m², with Reynolds numbers ranging from 6000 to 15,000 to capture the thermal-hydraulic performance across different flow regimes.

Flow rate was monitored using a calibrated digital instrument (DHYB-800) with an error tolerance of ± 0.15, while five thermocouples were positioned along the pipe wall to record surface temperatures. Two RTDs were installed at the inlet and outlet to measure bulk fluid temperatures. Pressure drop was determined using a precision gauge with an error tolerance of ± 0.08. All signals were acquired through a data recorder (AT47V), ensuring consistent and reliable measurements.

To maintain thermal equilibrium during continuous fluid circulation, a cooling unit and a heat exchanger were integrated into the closed-loop system. While hot fluids are often repurposed in practical applications, the indoor laboratory setup required active cooling to stabilize operating conditions. The working fluid was conditioned to ambient temperature prior to storage in the tank, and the laboratory environment was kept thermally stable throughout the experiments.

Measurements included wall temperature distribution, pressure drop, and flow rate for each tube configuration. These data, combined with the imposed boundary conditions and geometric parameters, were used to calculate the Nusselt number, friction factor, and the thermal-hydraulic performance factor (THPF).

Simulated solar radiation was applied at an intensity representative of realistic operating conditions^17^. Figure 4a and b illustrate the experimental layout.

#### Data reduction models

Once the boundary conditions are resolved, Eq. (7) facilitates the computation of flow velocity. This value subsequently serves as input for Eq. (8) to determine the Reynolds number, a pivotal dimensionless quantity that underpins nearly every aspect of the analytical evaluations conducted throughout this study^11^.

7 $$\:u = \:\frac{{\dot{m}}}{{\rho \:A}}$$

8 $$\:\mathrm{R}\mathrm{e}=\frac{{\uprho\:}{\mathrm{D}}_{\mathrm{h}}\mathrm{u}}{{\upmu\:}}$$

Following the determination of the Reynolds number, Eq. (9) enables the estimation of heat gain within the system. This thermal input serves as a foundational parameter for deriving the convective heat transfer coefficient via Eq. (10). Subsequently, Eq. (11) leverages these thermal characteristics to compute the Nusselt number, a key non-dimensional figure that plays a vital role in the thermal performance analysis central to this study^20^.

9 $$\:Q_{u} \: = \:\dot{m}\,CP\:(T_{{out\:}} - T_{{in\:}} )$$

10 $$\:{\mathrm{h}}_{\mathrm{i}}=\frac{\mathrm{q}{\prime\:}{\prime\:}}{{\mathrm{T}}_{\mathrm{w}\mathrm{a}\mathrm{l}\mathrm{l}}-{\mathrm{T}}_{\mathrm{r}\mathrm{e}\mathrm{f}}}$$

11 $$\:{\mathrm{N}\mathrm{u}}_{\mathrm{i}}=\:\frac{{\mathrm{h}}_{\mathrm{i}}{\mathrm{D}}_{\mathrm{h}}}{{\uplambda\:}}$$

To achieve an in-depth assessment of the tube’s heat transfer efficiency, parametric simulations were performed under varying flow conditions, relying solely on the Nusselt number is insufficient. It’s equally important to analyze the pressure drop and calculate f, as outlined in Eq. (12).

12 $$f = \frac{{2\Delta \:P}}{{\rho \:u^{2} }}\frac{{D_{h} }}{L}$$

At the final stage of evaluation, it becomes essential to weigh both Nu and the friction factor to understand how each contributes to improving the Thermal-Hydraulic Performance Factor THPF. Finding the right balance between heat transfer efficiency and flow resistance is key to identifying the most effective design. THPF, formulated as shown in Eq. (13), encapsulates this relationship and serves as a decisive metric. As demonstrated in^11^ THPF provides a balanced assessment framework for guiding conclusions regarding the thermal behavior of the tube.

13 $$\:THPF\: = \:\frac{{(Nu_{i} /Nu_{{ref)\:}} }}{{(f_{i} /f_{{ref}} )^{{\frac{1}{3}}} }}$$

#### Measurement uncertainty

All experiments are subject to two primary types of error, both of which must be identified to ensure that results can be reproduced within a predictable margin. The first is random error, typically caused by device inaccuracies, improper usage, or unsuitable methodologies. In this study, extensive care was taken to eliminate all sources of random error by thoroughly inspecting each component of the experimental setup. Despite this, the experiment remains susceptible to systematic errors that arise from inherent limitations in data recording across all measuring instruments. To account for this, Table 2 lists the percentage uncertainty associated with each device used in the study. Furthermore, the overall uncertainty for the experiment is quantified using Eq. (14), in accordance with reference^25^.

**Table 2:** Specified tolerances for instrumental Errors.

The devices of measuring	Brand	Boundary of error (±)
Resistance temperature probe	PT 100	0.15
Thermocouples	K-type	0.15
Flow meter (digital)	DHYB-800	0.5
Data recorder	AT47V	0.2
Water cooler	SPH20	0.2
Differential pressure gauge (digital)	DPG409	0.08

14 $$\:{\mathrm{W}}_{\mathrm{R}}=\:{[{\left(\frac{\partial\:\mathrm{R}}{{\partial\:\mathrm{X}}_{1}}\:{\mathrm{W}}_{1}\right)}^{2}\:+\:{\left(\frac{\partial\:\mathrm{R}}{{\partial\:\mathrm{X}}_{2}}\:{\mathrm{W}}_{2}\right)}^{2}+\:...\:{\left(\frac{\partial\:\mathrm{R}}{{\partial\:\mathrm{X}}_{\mathrm{n}}}\:{\mathrm{W}}_{\mathrm{n}}\right)}^{2}]}^{0.5}$$

$$\:{\mathrm{W}}_{\mathrm{R}}=\:{[{\left(0.2\right)}^{2}\:+\:{\left(0.15\right)}^{2}+\:{\left(0.15\right)}^{2}\:+\:{\left(0.5\right)}^{2}\:+\:{\left(0.08\right)}^{2}\:+\:{\left(0.2\right)}^{2}\:]}^{0.5}$$

$$\:{\mathrm{W}}_{{\mathrm{R}}} = 0.617\%$$

#### Validation of experimental result

**Fig. 5:**
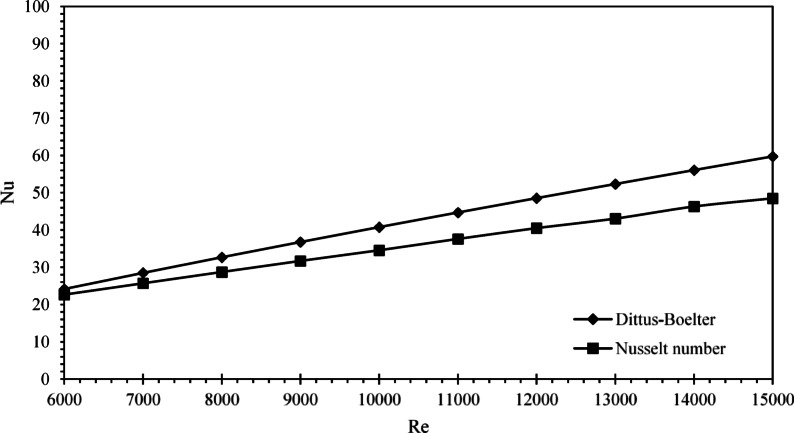
Comparison of experimental Nusselt numbers with Dittus-Boelter correlation predictions.

To assess the reliability of the experimental setup, the Nusselt number for water flowing through the circular tube was initially analyzed. The experimentally obtained Nusselt numbers were benchmarked against theoretical predictions derived from the Dittus-Boelter correlation. Figure 5 illustrates this comparison, highlighting the relationship between the Nusselt number and the corresponding Reynolds number, which ranged from 6000 to 15,000.

The Reynolds numbers of the tube’s interior flow, based on the established flow rates, were inside the totally turbulent range. The primary-side heat transfer coefficients were determined utilizing the Dittus-Boelter method^26^. Both the experimental data and the Dittus-Boelter model demonstrated a strong positive correlation between the Nusselt number and Reynolds number. The data exhibited an almost linear trend, with a correlation coefficient of 99.9%, indicating a high degree of consistency. Furthermore, the root mean square deviation (RMSD) between the experimental and theoretical values was found to be only 3.5%, underscoring the excellent agreement between the two datasets.

Despite minor deviations between the predicted and measured Nusselt numbers, the overall trend remained coherent. This consistency affirms the accuracy and robustness of the experimental methodology, validating its applicability for thermal performance analysis under the tested flow conditions.

## Results and discussion

### Taguchi-Based optimization results and performance interpretation

A total of 32 absorber tube configurations were systematically evaluated using a Taguchi L18 orthogonal array to examine the influence of fin geometry and flow parameters on convective heat transfer and overall thermo-hydraulic performance. Each configuration was defined by a unique combination of fin edge count, diameter, thickness, number of fins per rod, angular offset, and Reynolds number. Performance was assessed using two key indicators: the Nusselt number (Nu) and the thermal-hydraulic performance factor (THPF), as summarized in Table 3. The configuration 3E15D8F2MM40A10K, characterized by three fin edges, a tube diameter of 15 mm, eight fins per rod, a fin thickness of 2 mm, and an angular spacing of 40°, demonstrated the highest convective performance, achieving a Nusselt number of 133.17 at Re = 10,000. This superior thermal behavior is attributed to the combined effects of elevated fin density and pronounced angular separation, which enhanced boundary layer disruption and intensified vortex-induced mixing. Sharp fin protrusions facilitated localized flow detachment and reattachment, effectively breaking thermal stratification and promoting vigorous intermixing between hot and cold fluid regions. These flow phenomena are consistent with turbulence augmentation mechanisms reported by Sharaf et al.^19^ and Radhi et al.^27^ reinforcing the role of geometric optimization in improving convective heat transfer.

**Fig. 6:**
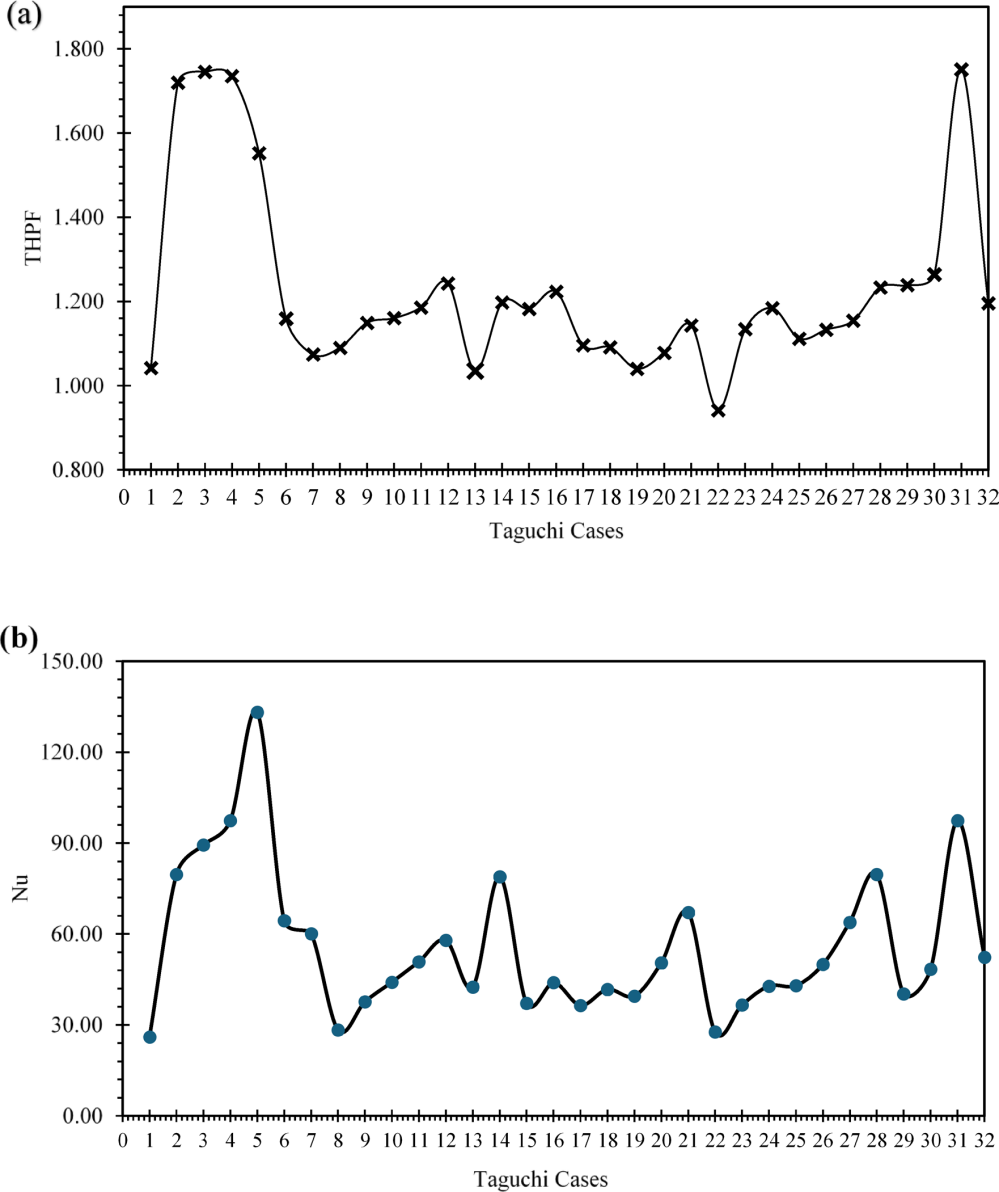
Multi-Objective Visualization of Taguchi Design Space: THPF Distribution and Nu-Re Response Surface (**a**) Chart showing the distribution of 32 Taguchi cases across THPF, (**b**) Impact of Geometric Parameters on Nu.

Based on Fig. 6a, Taguchi Case 3, 4, and 31 are the ones with the largest thermal-hydraulic performance factor (THPF), and, thus, confirm the high effectiveness of the corresponding cases of insert configurations compared to others. While Fig. 6b indicates, the Nusselt number has clear peaks at Taguchi Cases 5, 13, 21, and 30, which indicate high performance of convective heat transfer in these configurations.

Conversely, configuration 2E15D4F2MM0A6K exhibited the lowest thermal performance, with Nu = 26.07 at Re = 6,000. The limited fin count and absence of angular offset resulted in minimal flow disruption and weak turbulence intensity, leading to poor thermal exchange. From a thermo-hydraulic perspective, configuration 5E14.5D4F3MM0A8K emerged as the optimal design, achieving the highest THPF of 1.75 at Re = 8,000. Although its Nusselt number (97.40) was lower than that of the peak convective case, its streamlined geometry and reduced angular complexity minimized pressure losses, enabling superior overall efficiency. The lowest THPF was recorded in configuration 3E14D7F2.5MM10A6K, with a value of 0.941, confirming that insufficient turbulence and low Reynolds number not only limit heat transfer but also fail to justify hydraulic resistance.

These findings underscore the importance of integrating geometric tuning with flow optimization to avoid thermally and hydraulically inefficient designs. Figures 6a and b provide a visual summary of THPF and Nu across all Taguchi-generated configurations, serving as comparative benchmarking tools for rapid identification of high-performing geometries without exhaustive parametric sweeps.

**Table 3:** Taguchi cases numerical studies results.

No	Case name	Fin edges	Fin diameter	No.of fins	Thickness (mm)	Angle (‘)	Reynolds	Nu	THPF
1	2E15D4F2MM0A6K	2	15	4	2	0	6000	26.07	1.042
2	2E14D5F2.5MM10A7K	2	14	5	2.5	10	7000	79.69	1.720
3	2E14.5D6F3MM20A8K	2	14.5	6	3	20	8000	89.35	1.745
4	2E15.5D7F3.5MM30A9K	2	15.5	7	3.5	30	9000	97.42	1.735
5	3E15D8F2MM40A10K	3	15	8	2	40	10,000	133.17	1.552
6	3E14D9F2.5MM0A13K	3	14	9	2.5	0	13,000	64.42	1.159
7	3E14.5D4F3MM10A15K	3	14.5	4	3	10	15,000	60.10	1.074
8	3E15.5D5F3.5MM20A6K	3	15.5	5	3.5	20	6000	28.29	1.090
9	4E15D6F2MM30A7K	4	15	6	2	30	7000	37.64	1.149
10	4E14D7F2.5MM40A8K	4	14	7	2.5	40	8000	44.13	1.160
11	4E14.5D8F3MM0A9K	4	14.5	8	3	0	9000	50.85	1.186
12	4E15.5D9F3.5MM10A10K	4	15.5	9	3.5	10	10,000	57.96	1.243
13	5E15D4F2MM20A13K	5	15	4	2	20	13,000	42.50	1.035
14	5E14D5F2.5MM30A15K	5	14	5	2.5	30	15,000	78.97	1.198
15	5E14.5D6F3MM40A6K	5	14.5	6	3	40	6000	37.09	1.183
16	5E15.5D7F3.5MM0A7K	5	15.5	7	3.5	0	7000	43.96	1.223
17	2E15D8F2MM10A8K	2	15	8	2	10	8000	36.43	1.096
18	2E14D9F2.5MM20A9K	2	14	9	2.5	20	9000	41.74	1.091
19	2E14.5D4F3MM30A10K	2	14.5	4	3	30	10,000	39.54	1.040
20	2E15.5D5F3.5MM40A13K	2	15.5	5	3.5	40	13,000	50.47	1.078
21	3E15D6F2MM0A15K	3	15	6	2	0	15,000	67.11	1.144
22	3E14D7F2.5MM10A6K	3	14	7	2.5	10	6000	27.65	0.941
23	3E14.5D8F3MM20A7K	3	14.5	8	3	20	7000	36.60	1.134
24	3E15.5D9F3.5MM30A8K	3	15.5	9	3.5	30	8000	42.83	1.185
25	4E15D4F2MM40A9K	4	15	4	2	40	9000	42.96	1.112
26	4E14D5F2.5MM0A10K	4	14	5	2.5	0	10,000	49.95	1.133
27	4E14.5D6F3MM10A13K	4	14.5	6	3	10	13,000	63.90	1.154
28	4E15.5D7F3.5MM20A15K	4	15.5	7	3.5	20	15,000	79.68	1.233
29	5E15D8F2MM30A6K	5	15	8	2	30	6000	40.22	1.239
30	5E14D9F2.5MM40A7K	5	14	9	2.5	40	7000	48.36	1.264
31	5E14.5D4F3MM0A8K	5	14.5	4	3	0	8000	97.40	1.751
32	5E15.5D5F3.5MM10A9K	5	15.5	5	3.5	10	9000	52.37	1.195

**Fig. 7:**
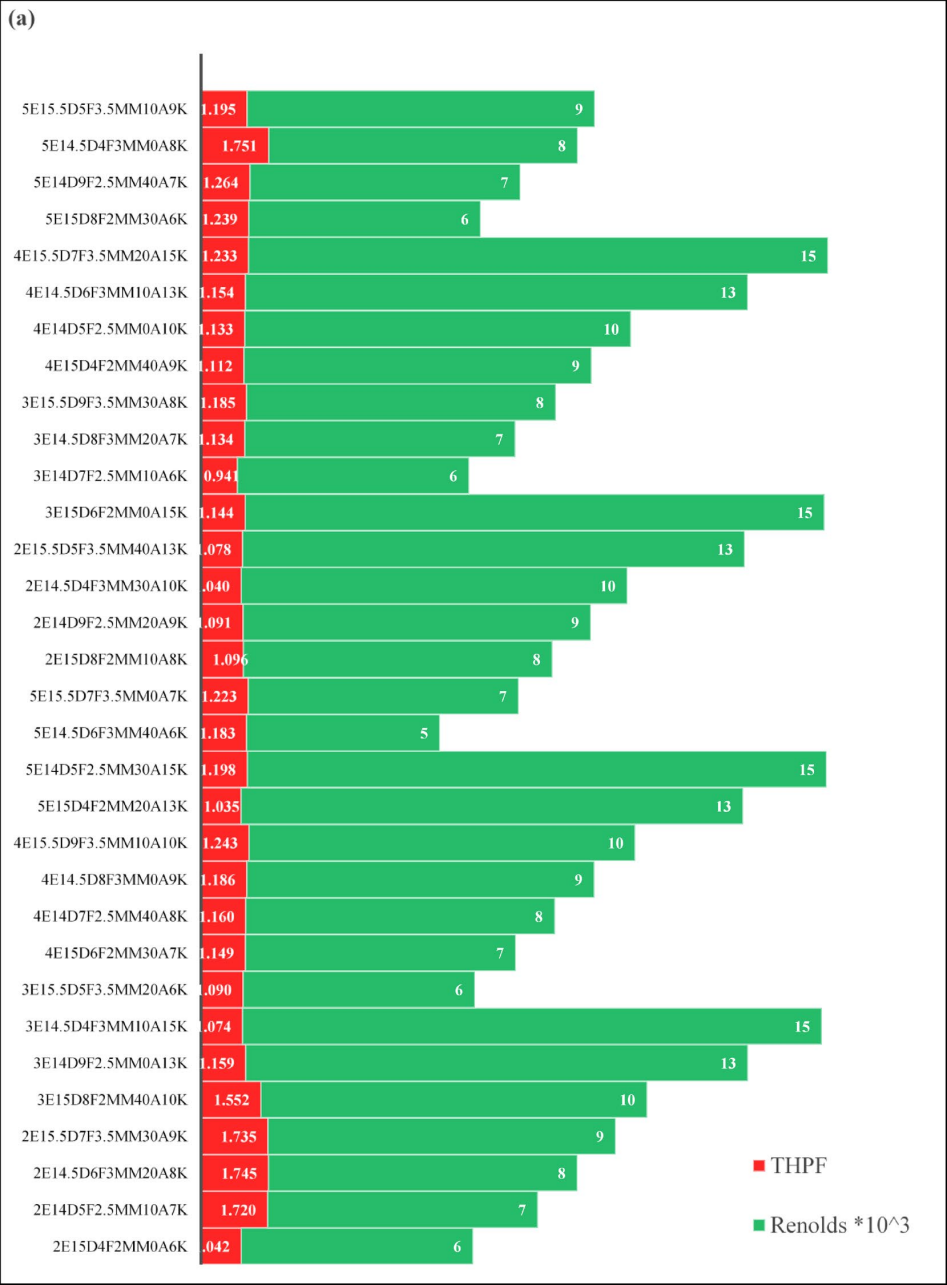

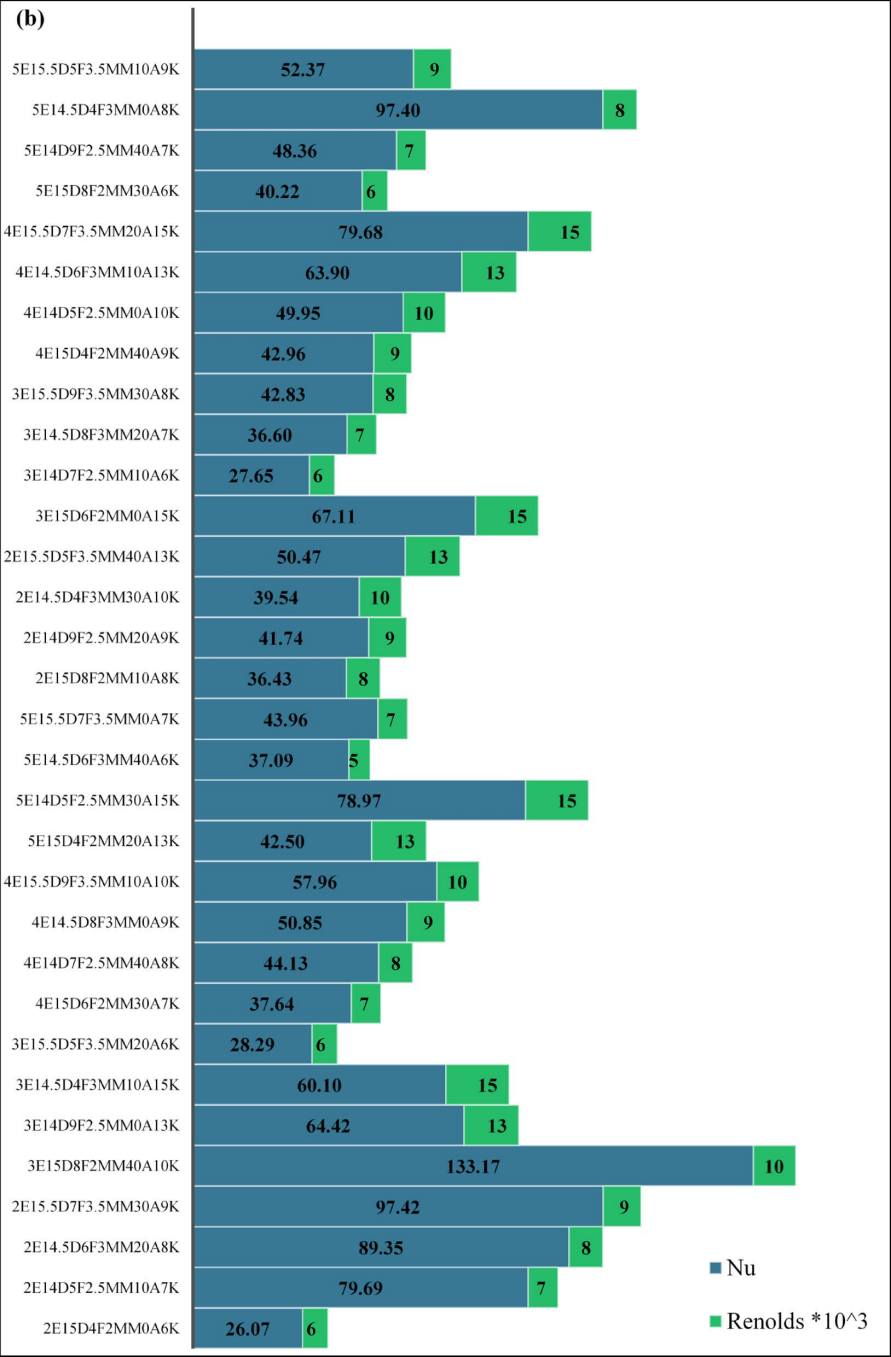

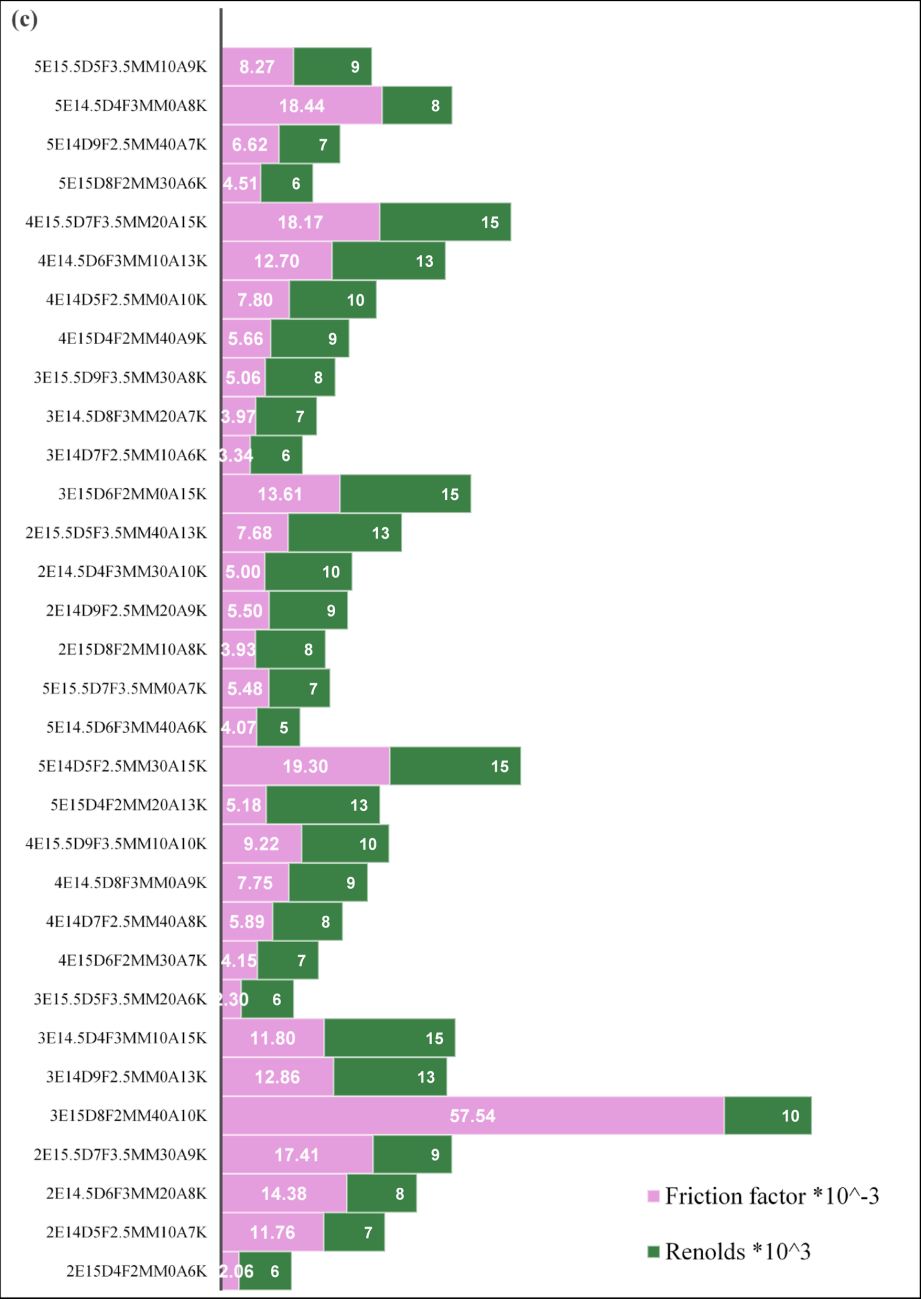
Thermal-hydraulic performance factor (**a**), Nusselt number (**b**), and friction factor (**c**) for simulated fin-core configurations with varying edge numbers.

Building on the Taguchi-based screening, a progressive selection of fin parameters was conducted to refine the optimal design. Figure 7a illustrates the variation of THPF across configurations. The comparative analysis reveals that Case 31 (5E14.5D4F3MM0A8K) achieved the highest THPF (1.751), representing the most favorable balance between heat transfer enhancement and hydraulic resistance. This superior performance is attributed to its optimized combination of five fin edges, moderate fin diameter (14.5 mm), relatively small number of fins (4), and zero angular offset. These geometric features promoted effective disruption of the thermal boundary layer and induced secondary flow structures, while avoiding excessive flow blockage. As a result, Case 31 delivered strong convective heat transfer (Nu = 97.40, Fig. 7 (b) without incurring prohibitive pressure losses, thereby maximizing overall efficiency.

In contrast, Case 5 (3E15D8F2MM40A10K) recorded the highest Nusselt number (Nu = 133.17, Fig. 7b, reflecting very strong heat transfer enhancement due to its high fin density (8 fins), large angular offset (40°), and relatively small fin thickness (2 mm). These parameters intensified vortex generation and mixing, elevating the convective heat transfer rate. However, the same geometric features also caused a substantial increase in flow resistance and friction factor. Consequently, the THPF for Case 5 was limited to 1.552, lower than that of Case 31, since the gain in heat transfer was partially offset by a greater pressure drop (Fig. 7c).

Overall, the results emphasize that maximizing heat transfer alone (as in Case 5) does not guarantee optimal thermo-hydraulic performance. Instead, the best configuration (Case 31) arises from a careful balance of fin geometry, number, and angular arrangement, which enhances convective transport while maintaining acceptable hydraulic losses.

### Flow structure and thermal stratification analysis

**Fig. 8:**
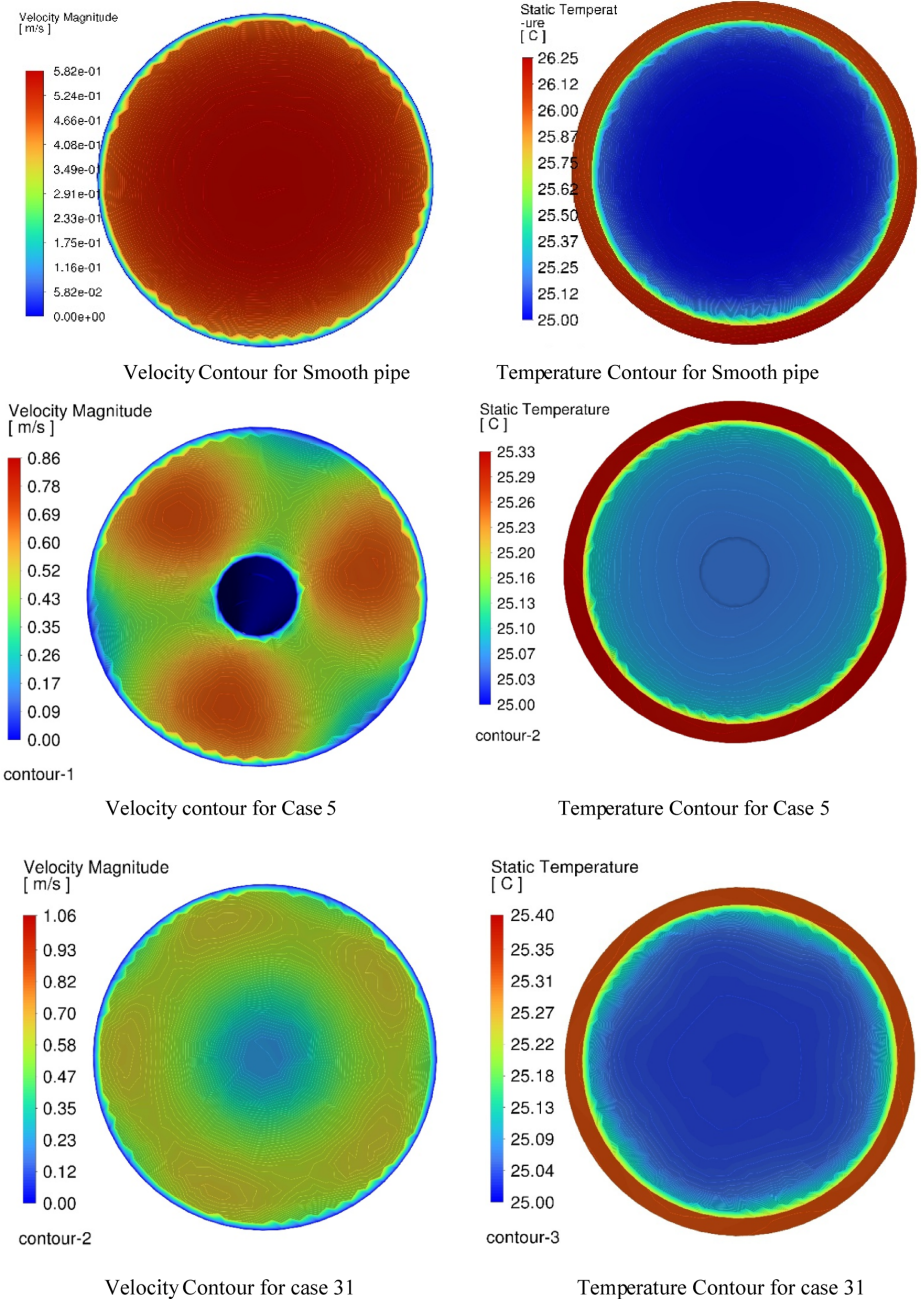

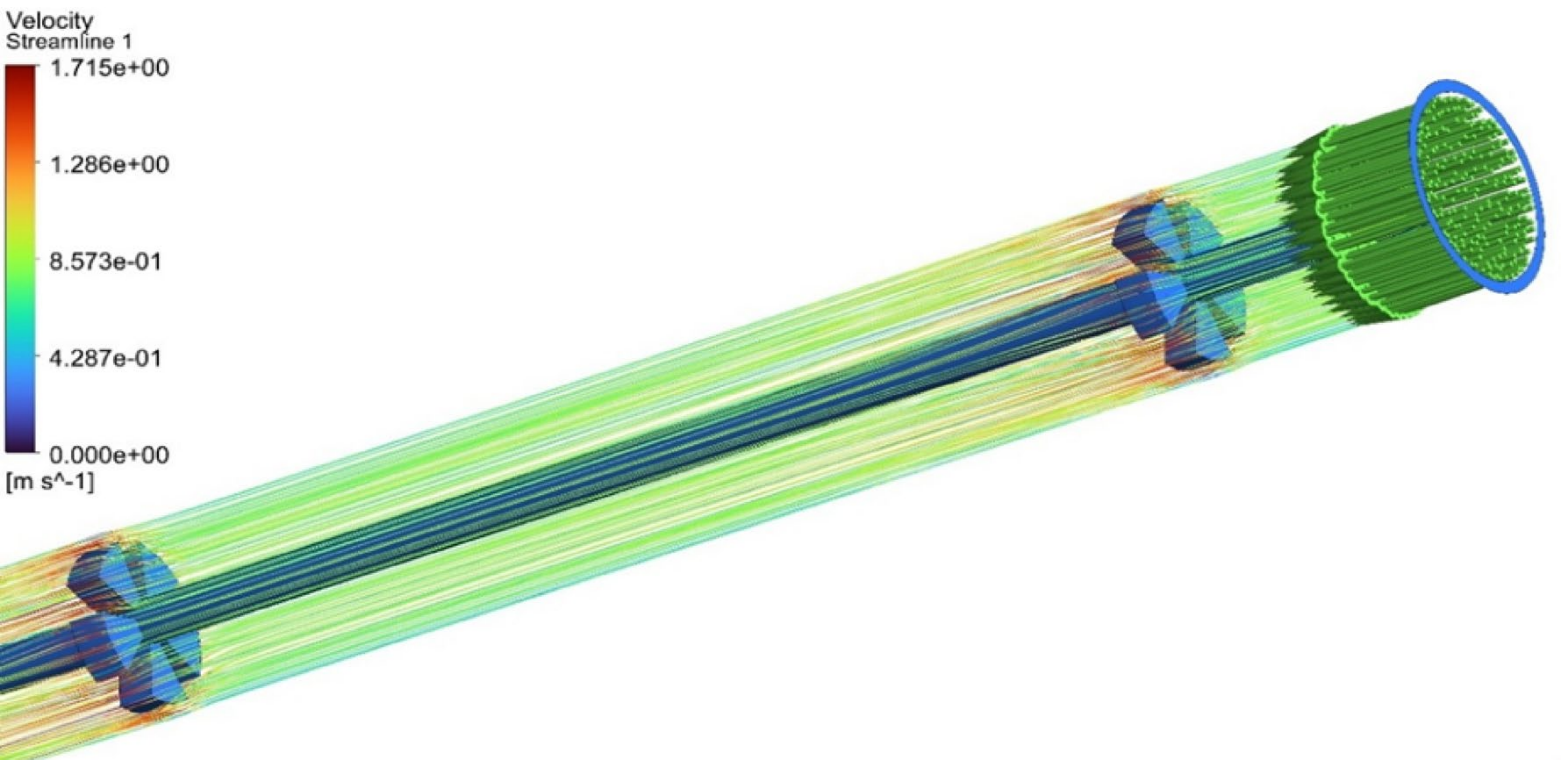
Comparative velocity and temperature contours for smooth and enhanced tube designs (Best Taguchi case 31 and case 5).

Figure 8 presents a comparative visualization of velocity and temperature contours for the smooth pipe, Case 5 (highest Nu), and Case 31 (highest THPF). This section analyzes the flow behavior and thermal stratification to clarify the physical mechanisms driving the observed performance metrics.

- Smooth pipe: The temperature distribution is relatively uniform, with the core remaining warmer and the wall region slightly cooler. This pattern is consistent with conduction-dominated heat transfer and limited mixing, resulting in modest thermal performance.
- Case 31: Distinct thermal gradients are observed, with localized cooling near the finned regions and elevated temperatures at the core. This stratification reflects enhanced thermal dispersion due to induced mixing, which facilitates heat transport from the wall to the core. The disruption of the thermal boundary layer reduces thermal resistance and improves overall convective efficiency.
- Case 5: The temperature field exhibits sharper gradients and more pronounced cooling zones, indicative of high heat transfer rates. However, the distribution is more chaotic, suggesting aggressive mixing and turbulent-like behavior. While this enhances thermal performance, it also contributes to increased pressure losses, diminishing the net thermal-hydraulic benefit.

- Performance trade-off analysis

The comparative results highlight a fundamental trade-off between heat transfer enhancement and hydraulic efficiency. Case 5 achieves the highest Nusselt number (Nu = 133.17), confirming its superior thermal capability. However, the associated rise in friction factor leads to a lower thermal performance factor (THPF), emphasizing the adverse impact of excessive mixing. Conversely, Case 31 demonstrates a more balanced configuration, combining effective heat transfer with reduced pressure drop, resulting in the highest THPF (1.75). This balance is particularly advantageous for applications where energy efficiency and pumping power are critical design constraints.

Figure 8 thus provides a comprehensive visual and physical comparison that underscores the role of vortex-induced mixing in shaping thermal performance. By including both Case 5 and Case 31 in the CFD visualization, the analysis captures the nuanced interplay between flow disruption and thermal dispersion. These findings reinforce the importance of optimizing internal geometries not solely for maximum heat transfer, but for overall thermal-hydraulic efficiency.

### Streamline visualization and thermal layer disruption

**Fig. 9:**
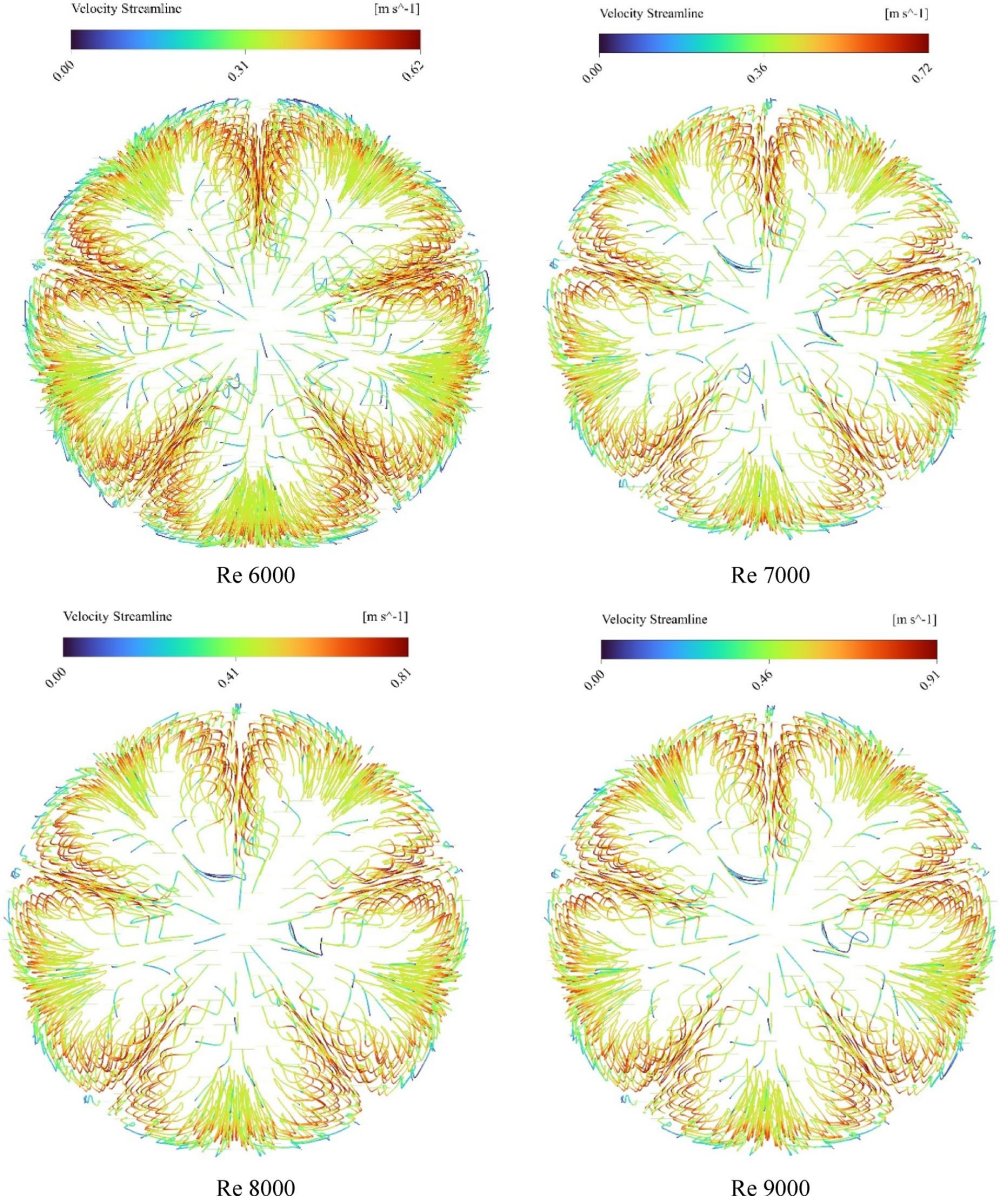

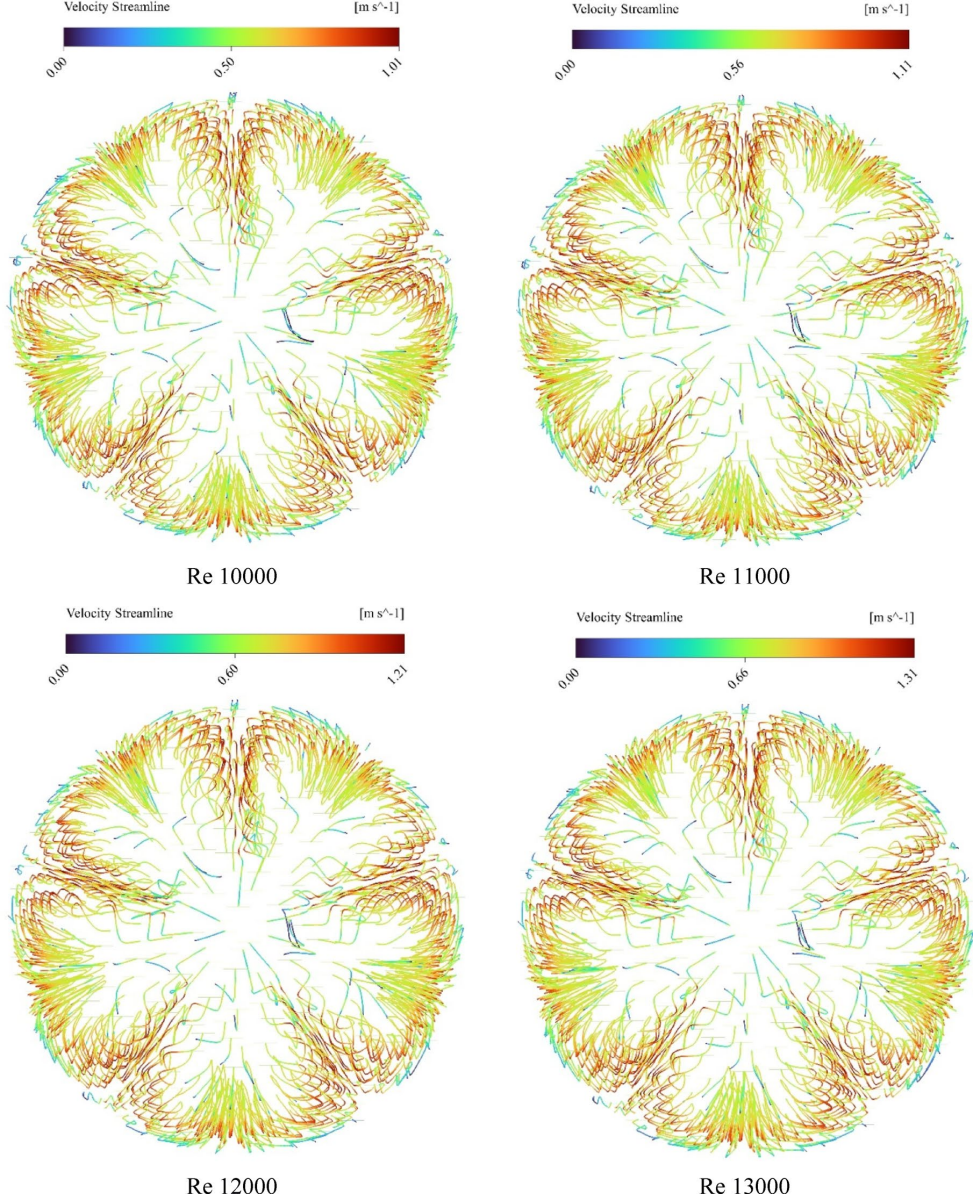

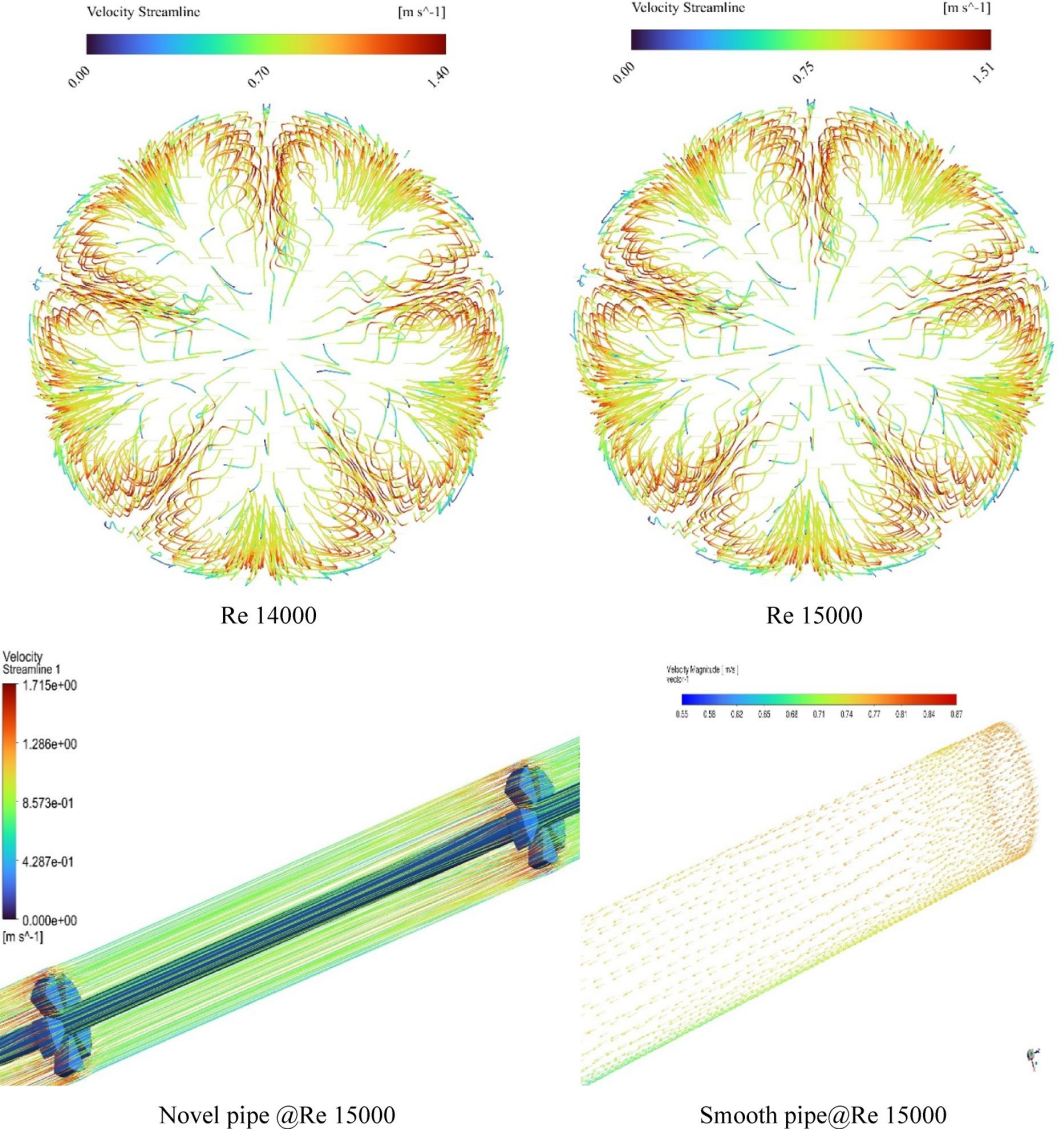
Velocity streamlines across reynolds numbers from 6000 to 15,000.

Figure 9 presents a CFD visualization of fluid streamlines within an absorber tube equipped with internal star-shaped fin structures. The streamlines are color-coded to represent velocity magnitudes, ranging from 0.314 m/s (deep blue) to 0.786 m/s (bright orange), as indicated by the accompanying legend. At the inlet, the streamlines begin with moderate velocity (blue to cyan), reflecting laminar entry conditions. As the fluid progresses through the finned section, the internal structures induce localized flow disturbances. These are evident in the form of streamline curvature, separation zones, and colour transitions from blue to green and yellow, indicating acceleration and enhanced mixing. Around the fins, velocity temporarily decreases due to obstruction and boundary layer interaction, followed by re-acceleration downstream, where streamlines shift toward warmer tones (yellow to orange). This behaviour suggests the formation of secondary flows and vortex structures that intensify convective mixing and momentum redistribution. Toward the outlet, the streamlines stabilize, exhibiting smoother paths and higher velocity gradients, confirming efficient momentum recovery and energy transport. The overall distribution highlights the role of geometric enhancements in promoting flow uniformity and improving thermal-hydraulic performance within the specified velocity range.

### Experimental validation and literature benchmarking

Table 4 presents a comparative synthesis of recent passive cooling techniques, including dimpled tubes, twisted tapes, micro-fin geometries, and hybrid enhancements, with reported THPF improvements ranging from 9% to over 70%. This benchmark provides a contextual foundation for evaluating the current study’s absorber tube configurations: smooth, Taguchi-optimized, and experimentally validated.

**Table 4:** Literature-Grounded comparison of thermal hydraulic performance Factor.

References & Year	Techniques	System description and main findings	THPF
Ref.^8^2024	Dimple shape	Improve the THPF	7.8%
Ref.^28^2024	Egg shaped	Including spherical, conical, and elliptical forms. This innovation enhances the thermal-hydraulic performance factor (THPF) by 12.5%. The chosen range (5000 ≤ Re ≤ 15,000) ensures fully turbulent flow conditions, which is critical for evaluating heat transfer and pressure drop in enhanced tube geometries.	12.5%
Ref.^29^2025	U-cut twisted tapes (U-TTs)	1.28 was achieved by U-TTs with an s/t ratio of 0.5 and a y/w of 3.5 at a Reynolds number of 6000.	28%
Ref.^30^2024	Dimple tube	Optimization of dimple geometry (diameter and pitch) under Re = 4000–15,000.	30%
Ref.^19^2024	Multi-spring wires insert	Flow turbulence and molecular agitation, resulting in a 174% increase in the Nusselt number	65%
^[Ref.15^2025	Serpentine collector with twisted tape and novel square spring	Varies solar irradiance (400 to 1000) W/m, the flow rate was from (0.0166 kg/s to 0.0498 kg/s)	37%
This study	Experimental absorber tube with Fins Insertor	Validated under Re = 6000–15,000; achieved Nu = 78.33	75%

**Fig. 10:**
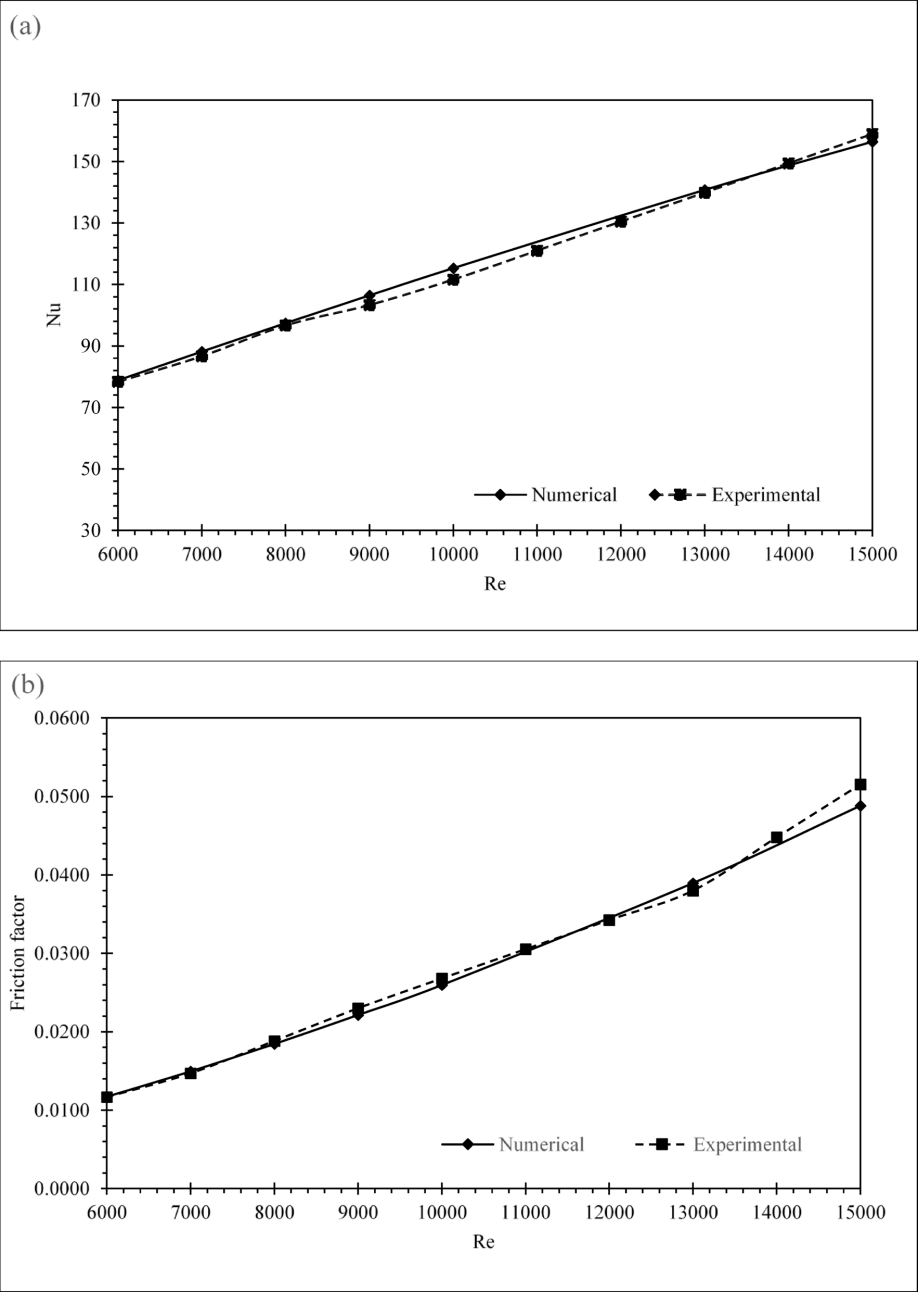

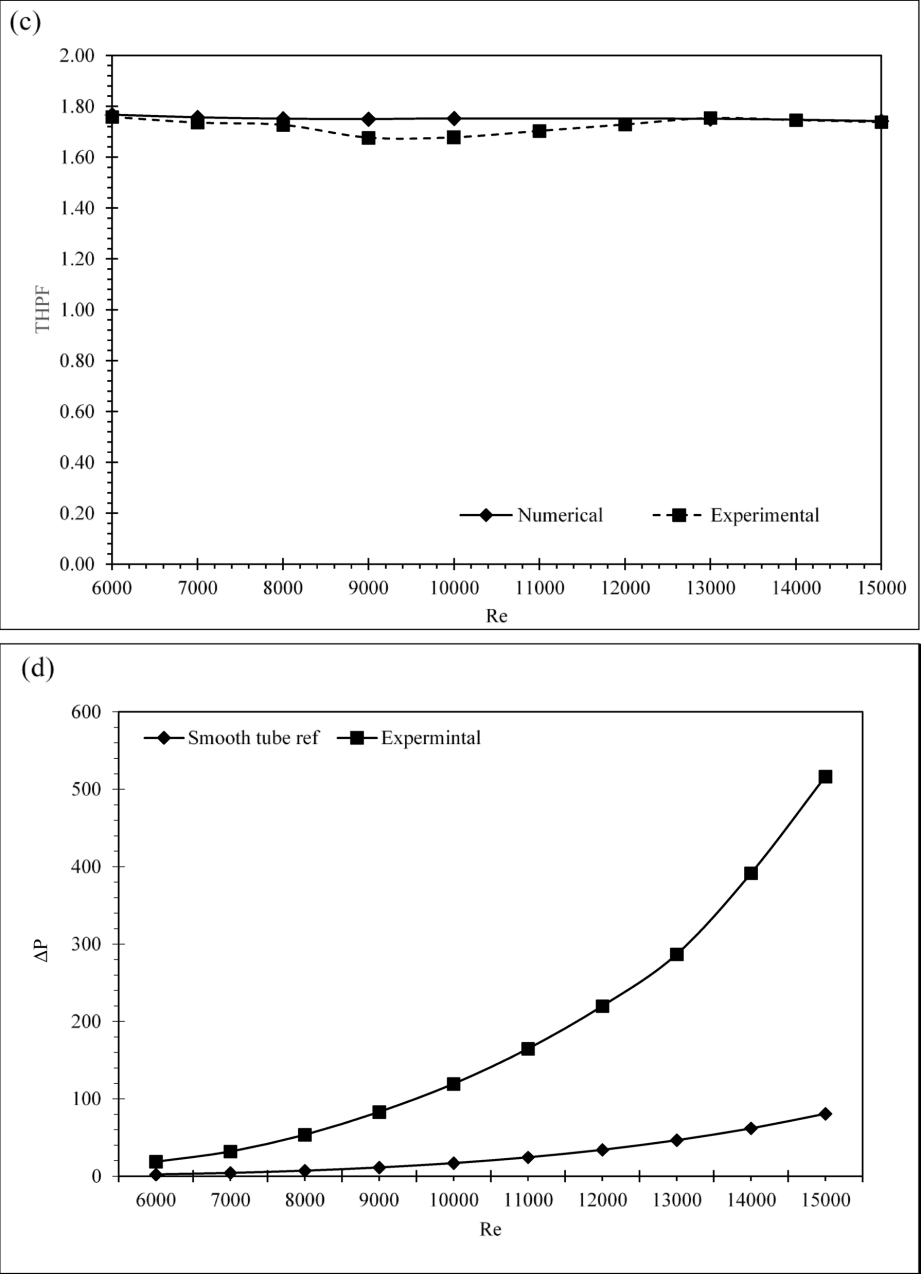
Comparative performance of absorber tube designs on experimental and Taguchi configurations based on: (**a**) Nusselt number, (**b**) friction factor, (**c**) THPF, and (**d**) Pressure drop.

The experimental design achieved a peak Nusselt number of 78.33 and THPF of 1.7583 at Re = 6,000, positioning it among the highest-performing geometries reported to date. This performance reflects the synergistic effect of optimized fin geometry and flow-induced vortex generation, as supported by the nonlinear growth trend in Nu with increasing Re. (Fig. 10a). Although the friction factor in Fig. 10b is higher than that of the smooth pipe, it remains within acceptable thermal optimization thresholds, The Taguchi-optimized configuration (Nu = 78.83, THPF = 1.7669) demonstrates a substantial enhancement over the smooth pipe baseline (Nu = 22.66, THPF = 1.0), confirming that the thermal improvement sufficiently offsets the associated increased pressure loss. Validating its relevance within the optimization landscape. Figure 10c illustrates the THPF distribution across Reynolds numbers, with the experimental case maintaining the highest values, followed closely by the Taguchi design, and the smooth pipe trailing behind. Numerical and experimental results showed strong agreement in Nusselt number and THPF across all Reynolds numbers, confirming the reliability of the CFD methodology. The optimized absorber tube consistently outperformed the smooth baseline and matched numerical predictions with high accuracy. In specific Reynolds ranges, its performance exceeded several published designs. This validates the robustness of the parametric framework and the effectiveness of geometric tuning. The proposed configuration offers high thermal efficiency with acceptable hydraulic penalties. It presents a scalable, manufacturable solution for next-generation PVT systems where modularity and energy performance are critical. The pressure drop behavior (Fig. 10d clearly shows the difference between the smooth tube and the one with the inserts, since it demonstrates the trade-off between augmented heat transfer and increased flow resistance. Figure 10a, b, c, and d illustrates a comparative analysis of simulated and experimental Nusselt numbers, friction factors, and THPF values assessing the accuracy of the numerical model within a Reynolds number range of 6000 to 15000. The trend demonstrates a consistent increase in Nu with Re for both datasets, corroborating the expected behavior of convective heat transfer under turbulent conditions. The numerical predictions closely correspond with the experimental results, exhibiting minor discrepancies at increased Reynolds numbers. The root mean square error (RMSE) was calculated to assess the discrepancy using the subsequent formula: The resulting RMSE was 4.5%, indicating strong agreement between the numerical and experimental results. This level of uncertainty is considered acceptable for turbulent flow models with complex geometries, especially when utilizing passive augmentation inserts.

## Conclusion

This research presents a detailed parametric and experimental investigation aimed at enhancing the thermo-hydraulic performance of a star-fin-integrated absorber tube, developed for passive cooling in thermal systems such as photovoltaic thermal (PVT) collectors. The investigation focused on five key geometric parameters: number of fin edges, fin diameter, fin thickness, number of fins per rod, and angular spacing. These variables were systematically optimized using a Taguchi orthogonal array, supported by MATLAB-based analysis and AutoCAD modelling. The tested ranges included 2–5 edges, 14.0–15.5 mm diameter, 2–3.5 mm thickness, 4–8 fins per rod, and 0°-40° angular spacing, with a fixed tube length of 550 mm to ensure consistency across all configurations. CFD simulations were conducted across Reynolds numbers from 6000 to 15,000. Among the tested designs, the configuration labelled 5E14.5D4F3MM0A, featuring five edges, a 14.5 mm fin diameter, four fins per rod, 3 mm thickness of fin, and zero angular offset, achieved the highest THPF of 1.754. This design demonstrated superior heat transfer due to increased surface area, controlled vortex generation, and uniform temperature distribution, as confirmed by contour plots and mesh convergence analysis. To validate the numerical findings, a physical prototype was fabricated and tested experimentally. The results showed a peak Nusselt number of 158.91 and a THPF of 1.7379 at Re = 15,000, with a deviation of less than 1.5% from the CFD predictions. The Realizable k-ε turbulence model with enhanced wall treatment proved effective in capturing near-wall gradients and complex flow behaviour. When compared with benchmark designs from the literature, the proposed absorber tube performed competitively and, in certain Reynolds ranges, exceeded previously published configurations. Although the friction factor was higher than that of a smooth pipe, the improvement justified the hydraulic burden. The Taguchi-optimized configuration (Nu = 156.43, THPF = 1.7419) significantly outperformed the smooth baseline (Nu = 48.50, THPF = 1.0), reinforcing the robustness of the parametric framework. These findings highlight the potential of the optimized absorber tube for scalable use in industrial thermal management and renewable energy systems. The integration of geometric tunability with passive enhancement strategies offers a reproducible and cost-effective pathway for improving energy conversion efficiency in PVT applications. The CFD model was validated against experimental data, achieving a correlation coefficient of 95.5% and a root mean square error (RMSE) of 4.5%, confirming the reliability of the simulation framework. The proposed configuration offers a scalable, manufacturable solution for compact heat exchangers, balancing thermal enhancement with hydraulic performance. By leveraging water as the working fluid and avoiding reliance on advanced materials or active energy inputs, the design ensures practicality and cost-effectiveness.

Future work should focus on expanding the optimization framework to include additional geometric and operational variables such as rod diameter, blade shape, pipe dimensions, and multi-variable interactions. Evaluating the thermal performance of the proposed design under elevated heat flux conditions would provide insight into its robustness and reliability. To ensure real-world applicability, outdoor experiments are recommended to validate system behaviour under fluctuating environmental conditions and varying solar irradiance. Additionally, investigating the effects of different nanoparticle types and concentrations in both nanofluids and nanoparticle-enhanced phase change materials (nano-PCMs) could help benchmark their contribution to thermal enhancement and guide material selection for future designs.

## Data Availability

The datasets generated and/or analysed during the current study are available from the corresponding author, upon reasonable request.

## Abbreviations

A: Area of the pipe (m^2^)
C_p_: Specific heat (J/Kg K)
D_h_: Hydraulic diameter (m)
$\:{f}_{i}$: Initial friction factor
$\:{ʄ}_{ref}$: Reference Friction factor
$\:{h}_{i}$: Initial convective heat transfer coefficient (W/m^2^K)
K: Thermal conductivity (W/m. K)
L: Length of the pipe (m)
ṁ: Mass flow rate (kg/s)
$\:{\mathrm{N}\mathrm{u}}_{\mathrm{i}}$: Initial Nusselt number
$$\:{Nu}_{ref\:}$$: Nu reference
$$\:\mathrm{R}\mathrm{e}$$: Reynolds number
$\:\mathrm{T}\mathrm{H}\mathrm{P}\mathrm{F}$: Thermal-hydraulic performance factor
$$\:{\mathrm{T}}_{\mathrm{i}\mathrm{n}\:}$$: Inlet temperature (K)
$$\:{\mathrm{T}}_{\mathrm{o}\mathrm{u}\mathrm{t}\:}$$: Outlet temperature (K)
$$\:{\Delta\:}\mathrm{P}$$: Pressure drops
$$\:\mathrm{u}$$: Velocity (m/s)
$\:{\upmu\:}$: Dynamic viscosity (Pa. s)
∅: Volume fraction (%)
K: Turbulent kinetic energy

## Greek symbols

$$\:{\uprho\:}$$: Density (Kg/m^3^)
$\:{\upvarepsilon\:}$: Specific heat dissipation rate
$$\:q{\prime\:}{\prime\:}$$: Heat flux
$$\:{\uplambda\:}$$: Thermal conductivity
